# The association of self-reported physical activity on human sensory long-term potentiation

**DOI:** 10.3934/Neuroscience.2021023

**Published:** 2021-05-24

**Authors:** Damien Moore, Paul D Loprinzi

**Affiliations:** Exercise & Memory Laboratory, Department of Health, Exercise Science and Recreation Management, The University of Mississippi, University, MS 38677, USA

**Keywords:** electroencephalogram, exercise, memory, long-term potentiation, synaptic plasticity

## Abstract

Exercise has been shown to enhance synaptic plasticity, therefore, potentially affecting memory. While the mechanism(s) responsible for this relationship have been explored in animal models, current research suggests that exercise may possess the ability to induce synaptic long-term potentiation (LTP). Most of the LTP mechanistic work has been conducted in animal models using invasive procedures. For that reason, the purpose of the present experiment was to investigate whether self-reported exercise is related to human sensory LTP-like responses. Nineteen participants (M_AGE_ = 24 years; 52.6% male) completed the study. Long-term potentiation-like responses were measured by incorporating a non-invasive method that assess the change in potentiation of the N1b component produced from the visual stimulus paradigm presented bilaterally in the visual field. Results demonstrated that those with higher levels of moderate-to-vigorous physical activity (MVPA) had a greater N1b change from baseline to the early time period assessment, *r* = −0.43, *p* = 0.06. Our findings provide some suggestive evidence of an association between self-reported MVPA and LTP-like responses. Additional work is needed to support that the potentiation of the human sensory N1b component in the observed study is due to the exercise-induced synaptic changes similar to that detailed in prior animal research.

## 1. Introduction

Engaging in acute and chronic physical activity is associated with improvements in overall cognitive performance [1]. Further, both acute and chronic exercise have been shown to enhance various cognitions, including memory function [2],[3]. At the cellular level, changes in synaptic plasticity as a function of exercise are thought to facilitate changes in the brain, leading to enhanced and sustained neural communication between neural components. Of interest, the phenomenon known as long-term potentiation (LTP) has been the subject of discussion and understood to be a potential driving force partly responsible for exercise-induced changes in synaptic plasticity, enhanced neural communication, and ultimately, improved memory performance. Specifically, LTP refers to the persistent strengthening of synapses that produces a long-lasting enhancement in neural communication between two neurons, as demonstrated by sustained excitatory post-synaptic potentiation.

Synaptic LTP is a central physiological process underlying synaptic plasticity that can be induced by tetanic stimulation at high frequencies or by associative pre- and post-synaptic stimulation [4]. A chief property of cellular LTP is input specificity, meaning, when a distinct pathway is potentiated, neighboring pathways that have not been stimulated by the induction protocol do not show potentiation [4]. Additionally, LTP and long-term depression (LTD) express Hebbian characteristics, such as *N*-methyl-D-aspartate (NMDA) receptor dependence, saturation,^1^ and longevity [5].^2^ Notably, however, LTP may occur through NMDA independent mechanisms [6]. Long-term depression occurs as a result of low frequency stimulation leading to a decrease in synaptic efficiency. Since LTP's novel discovery in 1973 [7], it has been studied extensively in animals at the molecular and cellular levels, primarily in the hippocampus [8],[9]. Furthermore, LTP has been shown to occur outside of the hippocampus, specifically in the visual cortex [10].

Animal models have demonstrated that exercise can enhance LTP [11],[12]. Relatedly, particularly in the motor cortex, there is also accumulating research in human samples showing that exercise can augment the response to experimentally-induced neuroplasticity paradigms [13],[14].

Although still in development, noninvasive methods for studying LTP in the human brain are starting to be integrated [15]–[17]. For instance, Teyler et al. [17] reported an increase in the amplitude of the visual evoked potential (N1b component) recorded over the occipital lobe by electroencephalogram (EEG). Likewise, Smallwood et al. [16] reported a significant increase (*p* = 0.004) in the late post N1b component in participants who self-reported physical activity levels as high-activity compared to those who self-reported that their physical activity was low. The N1b component is a late phase of the N1 complex that is a negative event-related potential (ERP) occurring ~170–190-ms post stimulus onset [18]. As such, potentiation of the N1b component denotes visually-induced plasticity in the human cortex, therefore, indirectly providing insight in the understanding of the neural underpinnings of memory. For more details on using non-invasive visual stimulation for LTP induction, the reader is referred elsewhere [19]. Notably, N1b alteration from visual stimulation has been shown to correlate with memory performance [20]. Using similar paradigms to those employed by Tyler et al., animal studies have observed convergent results in the visual cortex of rats [5],[21]. These visually-evoked N1b responses are thought to reflect LTP-like responses, as opposed to simply representing attentional processes. In support of these claims, Clapp et al. suggested that the potentiation of the visual evoked potential was exclusive to the visual cortex, the selective potentiation only occurred for the N1b component among the other visual evoked potentials, and the increased amplitude of the N1b component was sustained for over an hour [19].

In the present study, our primary goal was to assess the association of self-reported exercise on sensory LTP-like responses in the visual cortex of humans by means of rapid visual stimulation as indicated by changes in the amplitude of the N1b component. In addition to evaluating physical activity as a continuous variable, participants were distributed into two groups differing in self-reported levels of moderate-to-vigorous exercise (<150 minutes per week vs. ≥150 minutes per week). We hypothesize that individuals engaging in more exercise will produce a greater (LTP-induced) amplitude of the N1b component compared to less active individuals. If effective, this paradigm will be useful for future human experimentation that evaluates the extent to which experimentally-induced exercise influences LTP-like mechanisms in the human visual cortex. Thus, developing an effective human LTP model will play a key role in the field of integrative neuroscience.

## 2. Materials and methods

### 2.1. Subjects

Data within this manuscript originates from a previous publication [22] where we examined the effects of human visual sensory stimuli on N1b amplitude in young adults. Our current paper differs from the previous in that, for this paper, we evaluate the association of self-reported exercise on human sensory LTP-like responses.

Nineteen right-handed college men and women participated in the present study. All of which had normal or corrected vision. All participants were divided into two groups based on their self-reported exercise level. Informed consent was obtained from each participant. Due to the use of rapid visual stimulation, only participants who reported that they did not suffer from any neurological conditions, epilepsy, or migraine headaches were included. Further, participants were excluded if they exercised five hours before the visit, consumed caffeine three hours before the visit, were a daily smoker, were pregnant, took memory altering substances in the past month, were a daily alcohol drinker (1+ alcoholic drink/day if female and 2+ alcohol drinks/day if male), or had a concussion in the past 30 days. All procedures were approved by the University of Mississippi Institutional Review Board.

**Table 1.** Characteristics of the sample (N = 19).

Group	Does Not Meet PA Guidelines	Meets PA Guidelines
*N*	9	10
*N* Males	4	6
*N* right-handed	9	10
	*M* (±SD)	*M* (±SD)
Age (years)	23.71 ± 3.15	23.5 ± 2.64
Body mass index (kg/m^2^)	25.47 ± 4.86	25.11 ± 5.02
PAVS score	68.33 ± 48.73	342.0 ± 167.45

### 2.2. Physical activity vital sign questionnaire

The Physical Activity Vital Sign Questionnaire (PAVS) survey was used evaluate the number of minutes per week participants engaged in moderate-to-vigorous physical activity (MVPA). Participants were not asked to change their MVPA for this study, but rather, were asked to report their habitual MVPA levels. This instrument is meant to assess how much physical activity an individual performs per week and whether they meet physical activity recommendations. The questionnaire involves asking participants two questions, 1) “On average, how many days per week do you engage in moderate to strenuous physical exercise (i.e., like a brisk walk)?”; 2) “On average, how many minutes do you engage in exercise at this level?” Those engaging in at least 150 minutes per week of MVPA were classified as meeting exercise guidelines; otherwise, classified as not meeting exercise guidelines. This assessment has demonstrated evidence of validity [23],[24]. Notably, this self-report MVPA measure correlates with accelerometer-assessed number of days ≥30 bout-min MVPA (*r* = 0.52, *p* < 0.001) [23].

### 2.3. Apparatus

**Figure 1.**
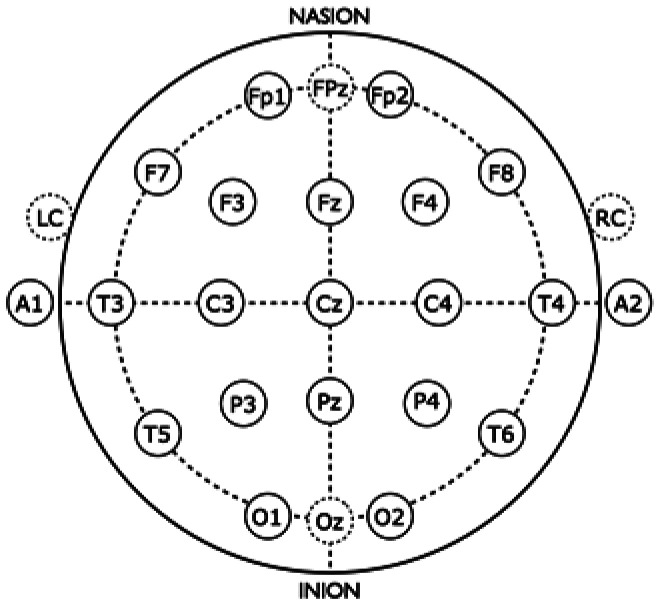
International 10–20 electrode placement.

As described in our previous publication [22], EEG data were collected using a NuAmps 40 channel, 22-bit, digital amplifier (Compumedics Neuroscan) with a fitted 32-channel Quik-Cap with integrated bipolar leads for vertical and horizontal eye movement. Quik-Caps were manufactured of highly elastic breathable Lycra material with soft neoprene electrode gel reservoirs for enhanced patient comfort. All electrodes were placed according to the International 10–20 electrode placement standard (Figure 1). For the present study, electrodes of interest included T5, P3, Pz, P4, T6, O1, Oz, and O2. Electrodes embedded in the Quik-caps were made with sintered Ag/Ag/Cl electrodes because of their durability and ease of cleaning and re-use. The NuAmps system utilizes SCAN 4.3 software for data acquisition software and STIM2 (Compumedics Neuroscan) software to time-lock visual presentations to collect EEG data. EEG recordings were sampled at a continuous 2000 Hz (0.1–100 Hz bandpass filter). Electrode impedances were below 30 kΩ, which is considered an acceptable level for this system [25]. A common vertex reference (Cz) was used to acquire EEG, which was later referenced to the average off-line.

### 2.4. Stimuli

As reported in our previous publication [22], and in alignment with other studies that have used a visual paradigm [16],[17], visual stimuli consisted of black and white reversal checkerboards presented bilaterally in the visual field. The stimuli subtended 4° of visual angle from the vertical and horizontal midline. The visual angle was computed from the size and the distance of the image from the observer. Visual angle calculation: A = (360/2π) (r/d) = 57.3(r/d), where A is the visual angle, pi (π) is approximately 3.14159, r is the size of the stimulus on the screen, and d is the distance of the observer from the screen. The stimuli were presented on a computer monitor (30-inch x flat-screen SVGA monitor with a resolution of 1920 × 1080 pixels Full HD at 60 Hz) 57 cm from subjects and checkerboard luminance was at 100 percent contrast. Checkerboards were generated by custom software Stim2 (NeuroScan INC) on a Dell Pentium II/200 computer. The transitor-transitor logic (TTL) provided synchronization of stimulus events with EEG acquisition.

### 2.5. Procedure

**Figure 2.**
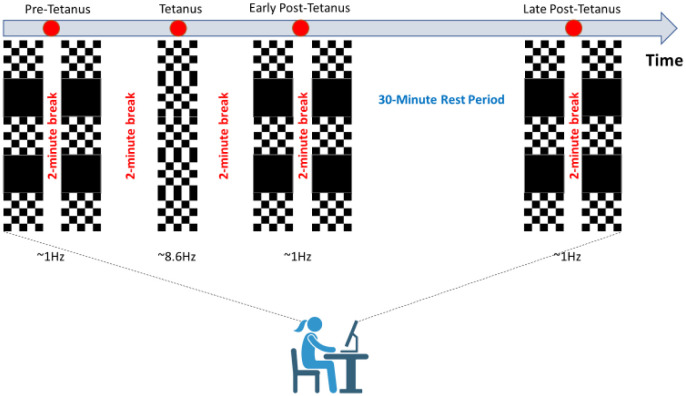
Visual stimulus paradigm. The pre-tetanus, early post-tetanus, and late post-tetanus blocks are comprised of interspersed presentations of 420 horizontal black and white reversal checkerboards presented for seventy seconds separated by fifteen seconds of a black screen with no checkerboards. During the pre-tetanus, early post-tetanus, and late post-tetanus blocks, the checkerboards were presented centrally at a rate of 1 Hz. The tetanus consisted of 1000 consecutive presentations of horizontal black and white reversal checkerboards presented centrally lasting for ~2-minutes. The pre-tetanus, early post-tetanus, late post-tetanus and tetanus blocks are separated by two-minute breaks (eyes closed) to allow for any retinal after-image to dissipate. A 30-minute rest period occurred between the early post-tetanus and late post-tetanus periods.

See Figure 2 for a schematic of the procedure, which consisted of five phases. First, two pre-tetanus blocks. Second, a photic stimulus. Third, two early post-tetanus blocks. Fourth, a 30-minute rest period. And fifth, two late post-tetanus blocks. Each pair of pre-tetanus, early post-tetanus, and late post-tetanus was separated by a 2-minute rest period with eyes closed. Moreover, after the photic tetanus, participants were instructed to close their eyes for two-minutes to allow any retinal after-image to dissipate. Participants, while resting their chin on a chin rest were required to fixate on a red circular dot in the center of the screen during data collection.

Each pre-tetanus, early post-tetanus, and late post-tetanus block lasted 10 minutes and comprised of interspersed presentations of 420 horizontal black and white reversal checkerboards presented for seventy seconds separated by fifteen seconds of a black screen with no checkerboards. During the pre-tetanus, early post-tetanus, and late post-tetanus blocks, the checkerboards were presented centrally at a rate of 1 Hz (stimulus-onset-asynchrony (SOA) range 950–1120-ms, duration 100-ms). A test frequency of 1 Hz was believed to be low enough not to have a physiological effect and not so long as to unnecessarily prolong the test blocks.

The photic tetanus consisted of 1000 consecutive presentations of horizontal black and white reversal checkerboards and lasted for ~2-minutes. Stimulus duration during the photic tetanus was 100-ms, with a randomly jittered inter-stimulus interval of 67–100-ms (temporal frequency: 8.6 Hz).

### 2.6. EEG data reduction

Electroencephalogram data for each participant was segmented with respect to event markers into 600-ms time epochs (100-ms before and 500-ms after stimulus onset) with epochs averaged by block (pre-tetanus, early post-tetanus, late post-tetanus). Epochs with eye blinks or artifacts were rejected and not included in the averaging process. The amplitude of the N1b component of the pre-tetanus, early post-tetanus and late post-tetanus visual evoked potentials were averaged for clusters of electrodes (T5, P3, Pz, P4, T6, O1, Oz, and O2) positioned over the left and right posterior aspect of the skull and the occipital cortex, respectively (Figure 1). Clusters of electrodes were chosen based on capturing accurate and reliable data derived from prior studies [15],[18]. In accordance with previously validated measures [17],[18], the amplitude of the N1b component was defined as the mean amplitude of the section of the evoked potential, extending from the peak of the N1 component to halfway between the N1 and P2 components. For each participant, LTP-like response was calculated by subtracting pre-tetanus N1b amplitude from early and late post-tetanus.

### 2.7. Statistical analysis

A mixed-factor repeated measures ANOVA (rmANOVA) was utilized to evaluate the changes in the N1b component amplitude over time and between groups from participants who met the exercise guideline (≥150 minutes per week) compared to participants who did not meet the exercise guideline (<150 minutes per week). Specifically, in this rmANOVA, the within-subject factor of Time and between-subject factor of Group were included. The main effects for Time (baseline N1b, early N1b, and late N1b), Group (meets exercise guideline, does not meet exercise guideline) and Time x Group interaction were evaluated. In addition to a rmANOVA, we computed Pearson correlation analyses to evaluate the association between MVPA (continuous variable) and changes in N1b from baseline to the early time period, as well as from baseline to the late time period. Statistical significance was established as a nominal alpha of 0.05. All analyses were computed in JASP (v 0.14.1.0).

## 3. Results

The current physical activity guidelines for adults (aged 18–64 years old) are at least 150 minutes of moderate-intensity aerobic physical activity, or at least 75 minutes of vigorous-intensity aerobic physical activity throughout the week [26]. In the present sample, the MVPA range was 0-135 min/week for the group that did not meet exercise guidelines compared to 180–720 min/week for the group that met exercise guidelines.

Table 1 displays the characteristics of the sample between the two exercise groups. The PAVS (physical activity) score for the group that did not meet exercise guidelines (*M* = 68.33 ± 48.73 minutes per week) was significantly lower than those meeting exercise guidelines (*M* = 342.00 ± 167.45 minutes per week), *t* (17) = −4.71, *p* < 0.001.

Among those not meeting MVPA guidelines, the mean (SE) N1b amplitude across the three respective time points was −0.04 (0.16), −0.68 (0.26) and −0.40 (0.24). These respective values for those meeting MVPA guidelines was −0.91 (0.29), −1.31 (0.40) and −1.13 (0.37). Results showed no significant main effect for Time, *F* (2, 34) = 2.911, ŋ_P_^2^ = 0.05, *p* = 0.07. However, and as reported in our previous publication [19], post-hoc analysis demonstrated that there was a statistically significant difference between baseline N1b (M = −0.498 µV, SD = 0.858) and early N1b (M = −1.011 µV, SD = 1.088), *t* (18) = 2.761, *p* = 0.039, d = 0.633.

**Figure 3.**
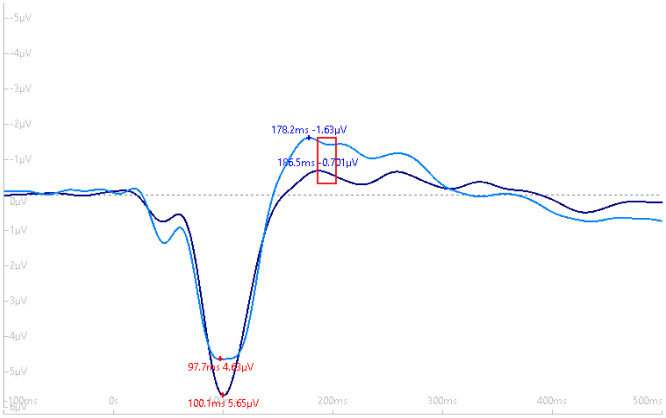
Grand-average waveform (with negative plotted up) demonstrating the difference in the N1b component amplitude (red box) between groups that met (light blue tracing) and did not meet MVPA guidelines (dark blue tracing).

**Figure 4.**
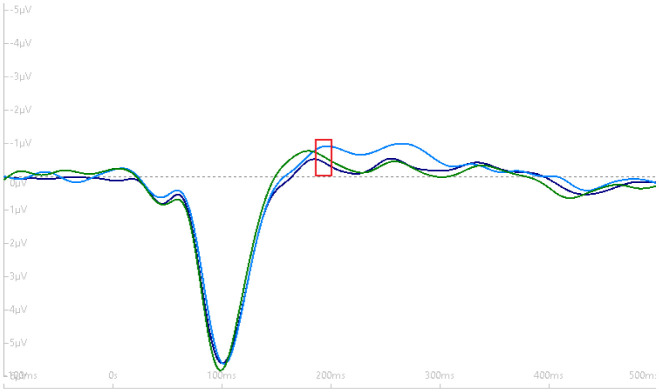
Within-subjects grand-average waveform (with negative plotted up) demonstrating no difference in the N1b component amplitude (red box) across the three time periods for the group not meeting exercise guidelines. Dark blue tracing (baseline N1b), light-blue tracing (early post-N1b) and green tracing (late post-N1b).

**Figure 5.**
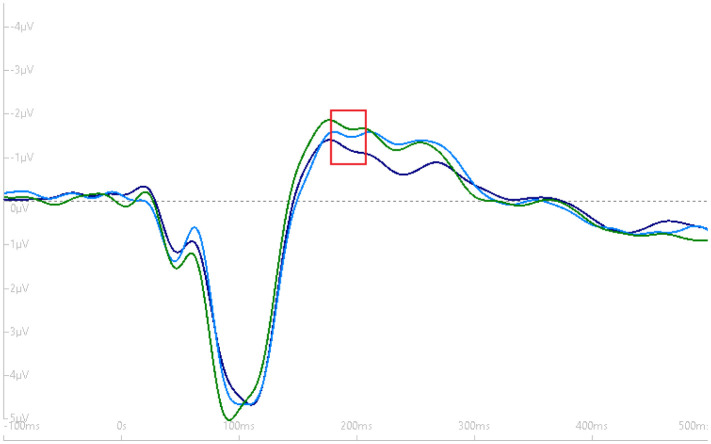
Within-subjects grand-average waveform (with negative plotted up) demonstrating no difference in the N1b component amplitude (red box) across the three time periods for the group meeting exercise guidelines. Dark blue tracing (baseline N1b), light-blue tracing (early post-N1b) and green tracing (late post-N1b).

We did not observe a significant Time x Group interaction, *F* (2, 34) = 0.15, *p* = 0.86. However, there was a marginal main effect for Group, *F* (1, 17) = 4.30, ŋ_P_^2^ = 0.14, *p* = 0.05. The mean group difference was, *M*_Difference_ = 0.74, SE = 0.36, (t) = 2.07, 95% CI: −0.01, 1.50, d = 0.48, *p* = 0.05. Results suggest that participants who met exercise guidelines (≥150 minutes per week) had a greater N1b amplitude compared to those who did not meet exercise guidelines (<150 minutes per week). Figures 3–5 depict the grand-average waveform and the N1b component differences between- and within-groups that met MVPA guidelines and those not meeting MVPA guidelines.

**Figure 6.**
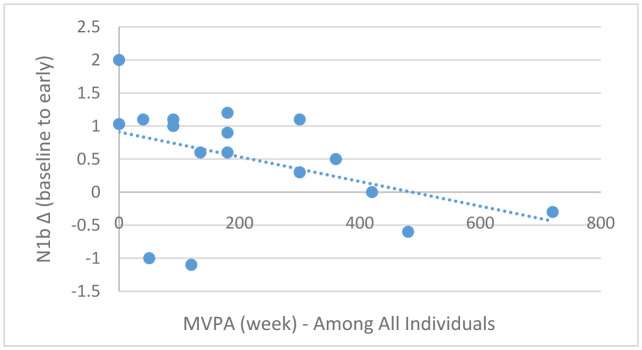
Scatterplot depicting the relationship between MVPA/week (x-axis) and baseline-to-early N1b changes (y-axis) among the entire sample, *r* = −0.43, *p* = 0.06.

**Figure 7.**
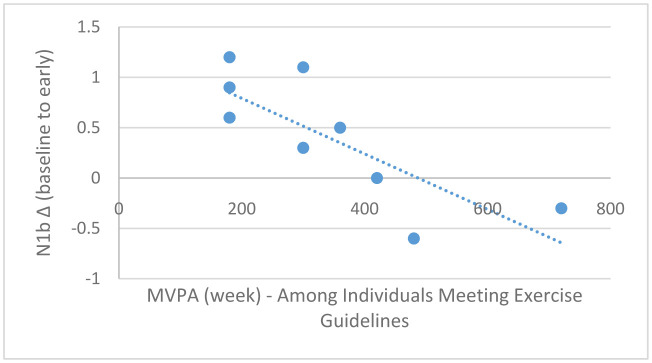
Scatterplot depicting the relationship between MVPA/week (x-axis) and baseline-to-early N1b changes (y-axis) among those meeting exercise guidelines, *r* = −0.78, *p* = 0.006.

**Figure 8.**
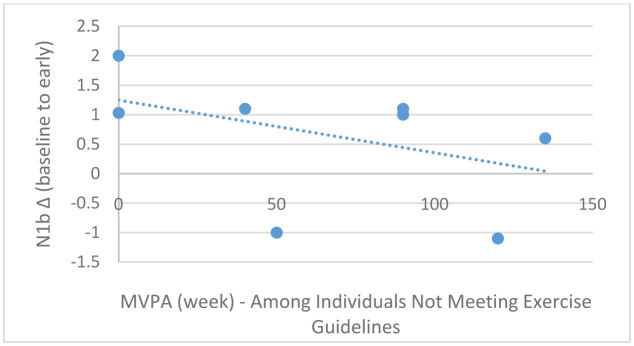
Scatterplot depicting the relationship between MVPA/week (x-axis) and baseline-to-early N1b changes (y-axis) among those not meeting exercise guidelines, *r* = −0.42, *p* = 0.25.

Pearson correlation analyses demonstrated a marginally statistically significant association between MVPA (continuous) and baseline-to-early N1b changes in the entire sample (*r* = −0.43, *p* = 0.06; Figure 6); this association, however, was stronger among those meeting exercise guidelines (*r* = −78, *p* = 0.006; Figure 7) compared to those not meeting exercise guidelines (*r* = −0.42, *p* = 0.25; Figure 8). Moderate-to-vigorous physical activity, however, was not associated with baseline-to-late N1b changes in the entire sample (*r* = 0.14, *p* = 0.58), as well as those meeting (*r* = 0.04, *p* = 0.89) and not meeting exercise guidelines (*r* = 0.48, *p* = 0.18).

## 4. Discussion and conclusions

The purpose of the current study was to assess whether self-reported exercise behavior is related to sensory LTP-like responses in the human visual cortex by means of rapid visual stimulation. Using the PAVS questionnaire, participants were distributed into two groups differing in the number of self-reported minutes per week engaged in moderate-to-vigorous exercise (<150 minutes vs. ≥150 minutes). Both groups, as a result of the tetanus stimulus, responded in a similar fashion by demonstrating an increase in the amplitude of the N1b component in the visual evoked potential.

Our main finding was that those who met exercise guidelines had a higher N1b amplitude across all time points, and further, those with higher levels of MVPA had a greater N1b change from baseline to the early time period. Previous research examining the influence of physical activity on N1b amplitude using a similar protocol have produced dissimilar findings. For example, Smallwood et al. [16] assessed the influence of physical activity on sensory LTP-like responses using the International Physical Activities Questionnaire (IPAQ) to classify participants as either less active or more active. Their results revealed a statistically significant main effect for time (*p* = 0.016) and a statistically significant interaction effect between time and group (*p* = 0.004). Yet, the main effect for activity level was non-significant (*p* = 0.102). In contrast, we observed a marginal main effect for group (*p* = 0.05) and a non-significant main effect for time (*p* = 0.07) with no group x time interaction (*p* = 0.86). In short, Smallwood et al. [16] showed that the LTP-like amplitude was greater for the early post-tetanus block compared to the late post-tetanus block, whereas we observed marginal differences in N1b amplitude across time periods. Moreover, N1b amplitude remained potentiated in the late post-tetanus block for the high activity group compared to the low activity group, whereas we showed active individuals had a higher N1b amplitude at all time periods and a greater change from baseline to early post-tetanus compared to less active individuals.

Notable discrepancies between studies could be from differences in the photic tetanus presentations and the assessment of physical activity. Our visual paradigm presented horizontal black and white reversal checkerboards bilaterally to both visual fields of the participants, whereas Smallwood et al. [16] presented circular horizontal and vertical sine gratings to participants. Lastly, the photic tetanus employed by Tyler et al. consisted of horizontal or vertical sine gratings (counterbalanced between participants), whereas our tetanus stimulus consisted of horizontal only presentations of black and white reversal checkerboards matching the orientation presented in the pre-, early-, and late post-tetanus blocks. Neurons in the occipital region of the brain are sensitive to stimulus orientation, and therefore, will become more active when a line of a particular orientation appears within its receptive field [27],[28]. Consequently, orientation-sensitivity of the neurons in the occipital region of the cortex may have exploited the difference between the two visual paradigms. Subsequently, Smallwood et al. incorporated the International Physical Activity Questionnaire (IPAQ) which utilized metabolic equivalents to group individuals into three levels of physical activity: low, moderate, and high. The PAVS questionnaire employed in our experiment assessed physical activity by the total number of minutes per week participants engaged in MVPA.

Self-reported exercise displayed noticeable difference in N1b amplitude between individuals who met exercise guidelines (≥150 minutes per week) compared to those not meeting exercise guidelines (*M*_DIFFERENCE_ = −0.74 µV, SE = 0.36, 95% CI: −1.50, 0.01, *p* = 0.05). Further, when MVPA was expressed as a continuous variable, those who were more active had greater changes in N1b from baseline to early. These results may suggest that engaging in MVPA allows for greater LTP-like responsiveness. We do, however, reserve caution in this interpretation. Although we observed a marginal main effect for group, as well as MVPA associating with baseline-to-early N1b changes, we did not observe a group x time interaction in the rmANOVA model. Further, the association between MVPA and changes in N1b from baseline to early may have been a result of a regression to the mean effect, as baseline N1b levels appeared to vary as a function of MVPA.

If exercise does, in subsequent work, demonstrate to be casually related to LTP-like responses, it is plausible that (exercise-induced) receptor saturation and longevity may play a role in LTP-like responsiveness, thus, influencing the amplitude of the N1b component as observed between groups measured at the baseline N1b timepoint. The greater number of postsynaptic receptors with increased sensitivity, saturation, and longevity, may allow for a greater influx of sodium and calcium ions, therefore, producing greater postsynaptic potentiation. Likewise, exercise has been shown to increase the production of brain derived neurotrophic factor (BDNF), which has been shown to enhance various stages of LTP; both mBDNF and proBDNF may play important roles in LTP and memory [29]. Previous research in animals supports these claims [1],[30], however, more research is required to confirm these findings in humans.

Limitations of our study include exercise behavior being self-reported as opposed to measuring exercise via a device (e.g., accelerometer). Utilizing a direct and accurate measurement of exercise and/or physical fitness may provide an in-depth understanding of how exercise influences changes in LTP-like responses when differences are observed between active and non-active participants. Additionally, diverse modalities of exercise, such as aerobic physical activity and resistance training, may influence changes in neuroplasticity and synaptic communication through similar and unique mechanisms [31]–[33]. Further, we recognize that the sample is small for a 2 (group) × 3 (time) experimental design study. Therefore, interpretation of the data should be approached with caution as we may have been underpowered to observe certain effects. Thus, future studies should incorporate a larger sample size in addition to a more robust measure of physical activity. Additionally, it is important to note that our study does not provide any mechanistic evidence that the observed phenomenon is LTP, but rather, only provides some evidence suggestive of it. Lastly, another notable observation of our study is that, although P100 did not differ across the time periods, there was a difference in P100 between those meeting and not meeting exercise guidelines, suggesting that attentional cost may vary as a function of exercise.

In summary, and as we demonstrated in our earlier publication [22], our findings suggest that it is possible to induce an LTP-like response in the visual cortex of humans non-invasively. Subsequently, and as shown herein, meeting exercise guidelines may, potentially, influence N1b amplitude. Additional work is needed to corroborate that the potentiation of the N1b component observed in this study is due to similar mechanisms essential to LTP induction in the cognitive structures of the brain observed in prior animal research. If so, this will allow for the examination of LTP-like responses and its interaction with multiple human sensory stimuli and behaviors.

## Footnotes

1 NMDA receptor saturation is a period in which all receptors are successfully occupied with the neurotransmitter glutamate.

2 NMDA receptor longevity reflects the length of time the ligand is bound to the receptor(s) before the ligand disassociates, reducing the activity/function of the receptors.
